# An evaluation of the effect of tube potential on clinical image quality using direct digital detectors for pelvis and lumbar spine radiographs

**DOI:** 10.1002/jmrs.403

**Published:** 2020-06-03

**Authors:** Nicole E Peacock, Adam L Steward, Peter J Riley

**Affiliations:** ^1^ Department of Medical Imaging Western Health Footscray VIC Australia; ^2^ School of Medicine, Faculty of Health Deakin University Waurn Ponds VIC Australia

**Keywords:** Radiation Dosage, Radiation Ionising, Radiographic Image Enhancement, Radiography, X‐Rays, kVp, Radiographic Image Quality

## Abstract

**Introduction:**

High kVp techniques, 15% or 10‐kVp rules, are well‐known dose reduction methods. Traditionally, the use of high tube potential (i.e. increased kVp) is associated with decreased radiographic contrast and overall image quality. Recent studies suggest contrast and image quality are not heavily reliant on kVp with digital systems. This study aims to assess the effects of the high tube potential technique on clinical radiographic image quality when using digital systems, to validate high kVp as a dose saving technique.

**Methods:**

A selection of comparable pelvis and lumbar spine radiographs were collected from the hospital’s picture archiving and communication system (PACS), with technical factors recorded. All clinical radiographs were assessed by 5 senior radiographers using a 15‐point visual grading analysis (VGA) rubric.

**Results:**

For 40 AP pelvis radiographs and 40 lateral lumbar spine radiographs, reduction in the dose area product (DAP) with higher kVp is seen. Average pelvis DAP at 75 kVp = 14.06 mGy.cm^2^; 85 kVp = 7.47 mGy.cm^2^. Average lumbar spine DAP at 80 kVp = 15.76 mGy.cm^2^; 90 kVp = 14.83 mGy.cm^2^. Image quality and contrast scores showed no statistically significant difference between the high and low kVp groups (z = 0.06 and 0.12, respectively). Average pelvis VGA score at 75 kVp = 11.26; 85 kVp = 12.55. Average lumbar spine VGA score at 80 kVp = 9.23; 90 kVp = 10.64.

**Conclusions:**

The high tube potential techniques allowed for reduced patient radiation doses whilst showing no degradation of diagnostic image quality in a clinical setting. This study successfully validates the high kVp technique as a useful tool for reducing patient radiation doses whilst maintaining high diagnostic image quality for digital pelvis and lumbar spine radiography.

## Graphical abstract

This study allowed for quantifiable assessment of the effect of increasing kVp values on radiographic image quality and assessment of patient radiation doses in a clinical setting. The results supported the hypothesis that increasing kVp values will not significantly reduce image quality, as no statistically significant change was determined in the image quality scores of the high and low kVp groups for each projection. This study successfully validates the high kVp technique as a useful tool for reducing patient radiation doses whilst maintaining high diagnostic image quality.

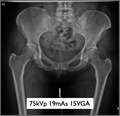

## Introduction

Diagnostic radiology examinations involving ionising radiation carry an element of risk and the potential to cause harm. In order to minimise these risks and ensure patient safety, radiation exposures used during radiological examinations should be kept ‘as low as reasonably achievable’ (ALARA).^1^ Exposure factors set by clinical radiographers at the x‐ray console for each examination directly impact the resultant patient radiation dose; therefore, optimisation of exposure factor selection is paramount. With the introduction of direct digital imaging systems, radiographers are no longer limited by the need to achieve a fixed film‐screen optical density and can therefore now manipulate exposure factors to achieve image quality and patient dose optimisation. There are a number of optimisation techniques described in the literature, including both high and low kVp techniques. High tube potential, or ‘high‐kVp’ techniques, which were established using traditional film‐screen technology, are well‐known and widely documented methods of reducing patient radiation doses.^2^, ^3^, ^4^, ^5^, ^6^, ^7^ The high kVp concept for dose reduction has since been carried over to digital imaging. The ‘15%’ and ‘10‐kVp’ rules are used to govern these techniques, stating that an increase in tube potential, measured as kilovoltage peak (kVp), by 15% or 10‐kVp requires the milliamperage seconds (mAs) value (which indicates the product of the tube current and exposure time) to be halved in order to keep the detector dose constant.^2^, ^3^, ^4^, ^8^, ^9^, ^10^ There are a large number of studies in the literature that confirm high kVp, low mAs techniques are effective in reducing patient radiation doses with digital imaging systems.^8^, ^9^ ^,^ ^11^, ^12^, ^13^, ^14^, ^15^, ^16^, ^17^ These dose saving abilities, however, are not infinite; some studies show that as the kVp reaches higher values (i.e. above 100 kVp), the dose reductions decrease and eventually taper off. In their 2014 study, Reis *et al*.^13^ showed a dose reduction of 18% when the kVp was increased from 80 to 90 kVp but only a 2% dose reduction when the kVp was increased from 100 to 110 kVp. This suggests there is a limit to how far the high kVp phenomenon can extend.

Traditionally, when film‐screen systems were used, an increase in kVp meant a loss of image contrast and overall image quality.^1^, ^4^, ^5^, ^7^, ^8^, ^16^ Currently, there is disagreement within the literature about how kVp selection effects image quality and contrast on digital imaging systems. Some sources, including Bushong’s textbook ‘Radiologic Science for Technologists: Physics, Biology and Protection’,^2^ state that kVp has no effect on resulting image contrast.^2^, ^6^, ^18^, ^19^ Other studies disagree, with some finding reduced contrast and image quality ^1^, ^5^, ^6^, ^7^, ^9^, ^20^ and others, improved image quality with higher kVp values.^4^ Low kVp techniques have also been described, as a method of image quality optimisation through exploiting the wide dynamic range of digital flat‐panel detectors. As previously described, with modern direct digital imaging systems, radiographers are no longer limited by the need to achieve a fixed optical density and therefore are able to increase the detector dose for improved signal to noise ratios, whilst keeping patient radiation doses the same, by reducing kVp values. ^3^, ^4^, ^8^ With these differing dose/image quality optimisation techniques and conflicting information seen in current published literature, questions are raised about the validity of such techniques and the best methods for optimisation. As clinical radiographers attempt to balance the trade‐off between radiographic image quality and patient radiation doses, further investigation into these optimisation techniques is required to determine which technique is most beneficial in which specific clinical situation.

## Aims and Objectives

The aim of this retrospective audit study was to investigate the effectiveness of the high‐kVp technique in a clinical setting with direct digital radiographic systems with flat‐panel detectors, for the reduction of patient radiation doses whilst maintaining diagnostic image quality for pelvic and lumbar spine imaging. The objectives were to:

- Quantifiably assess the effects of increasing kVp values on radiographic image quality when the detector dose remains constant
- Assess the dose saving capabilities of high kVp techniques in terms of patient radiation doses
- Use real, clinical pelvic and lumbar spine radiographs for evaluation to ensure results are relevant and applicable to clinical radiographers.

Previous research has shown pelvis imaging to have significant patient dose saving results when using high kVp techniques.^6^ AP pelvis projections were included in this study to ensure dose savings associated with using higher kVp values do not cause significant degradation to the resulting image quality. Lateral lumbar spine projections were also included as a relatively thick body region, prone to reduced image quality from scatter radiation, to ensure a higher kVp technique would not result in undiagnostic image quality.

## Hypothesis

With increased kVp values and fixed detector exposures, the authors postulated that:

- Radiographic image quality would not be significantly compromised
- All images would remain diagnostically acceptable
- Patient radiation doses would be reduced.

## Methods

Ethical approval was sought and granted for this retrospective study by Western Health’s Low Risk Ethics Panel in July 2018.

### Imaging systems

All radiographs included in this study were acquired at Western Health using the department’s GE Optima XR656 Direct Digital Radiographic Units with ‘Flash‐Pad’ Flat‐Panel Wireless Digital Detectors, a single panel amorphous silicon detector with a Cesium Iodide scintillator (GE Healthcare, Chicago, USA).

### Data collection

The study was designed as a retrospective analysis of existing radiological image data on Western Health’s Fujifilm Synapse picture archiving and communication system (PACS) (Fujifilm Medical Systems, Stamford, USA). Antero‐posterior (AP) pelvis radiographs and lateral lumbar spine radiographs were analysed over time periods of 3 and 6 months, respectively (from October 2017 to March 2018). A total of 217 AP pelvis images and 91 lateral lumbar spine images were available on Western Health’s PACS within these time frames. Technical information that was stored on PACS was recorded for each radiograph that met the inclusion criteria, including kilovoltage peak (kVp), milliamperage/sec (mAs), deviation index (DI) and dose area product (DAP).

### Inclusion criteria

As a retrospective study, the authors followed a strict inclusion criteria to ensure comparable radiographs and patient type (i.e. thickness) were being analysed. The assumption was made that patient thickness should be relatively consistent across radiographs generated using the same kVp if there was no more than 25% variation in the mAs and no greater than 1.0 difference in the deviation index (DI)**.** DI is a GE measurement of exposure, representing the variation between the actual exposure index (EI) and the target EI, with the base 10 logarithmic scale. EI is a measure of the radiation reaching the detector and, when divided by 100, is comparable to the detector entrance dose measurement in the unit µGy (when calibration conditions are used). The target EI varies depending on body region, projection and patient size selected. The EI, and subsequently the DI, is calculated through a complicated calculation workflow that takes into account anatomy (through an anatomic region identification algorithm), kVp/mAs, filtration, grid, receptor, speed, detector sensitivity and median image count; a further description of which is beyond the scope of this paper. A DI of 0 is optimal, with the optimal range of DI values between −3 and +2.^25^

The first inclusion criterion was that the kVp used to acquire the radiograph had to match either a low or high kVp value. These specific values were set after reviewing trends in exposure selection over the given time periods of data collection. AP pelvis images needed to be acquired at 75 or 85 kVp and lateral lumbar spine images at 80 or 90 kVp. The mAs values used for acquisitions needed to fall within set parameters, with the mAs of the higher kVp group deliberately set to half that of the lower kVp group, in keeping with the ‘10‐kVp’ rule described previously. For AP pelvis radiographs acquired at 75 kVp, 20 mAs ∓ 25% was set as the inclusion range. For AP pelvis radiographs acquired at 85 kVp, 10 mAs ∓ 25% was set as the inclusion range. For lateral lumbar spine radiographs acquired at 80 kVp, 80 mAs ∓ 25% was set as the inclusion range. For lateral lumbar spine radiographs acquired at 90 kVp, 40 mAs ∓ 25% was set as the inclusion range. The last inclusion criterion was for the radiograph to have a DI value falling between −0.5 and + 0.5. These assumptions on exposure selection and patient size are based on clinical assumptions made at the console by radiographers and are in keeping with the clinically based focus of this study. They do however present a limitation of the study, which is described in detail in the discussion.

### Exclusion criteria

**Figure 1:**
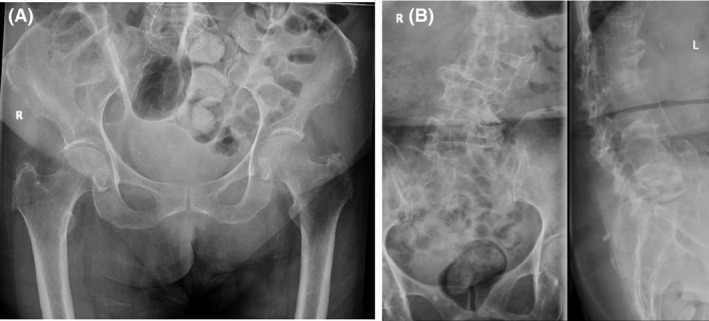
Examples of excluded radiographs (A) Gross overlying bowel gas (B) Severe scoliosis. An exclusion criteria were used to exclude radiographs such as these from this study, with overlying artefact or pathology obscuring visualisation of landmarks used for VGA scoring to prevent these negatively influencing VGA scores.

No paediatric images were included in this study. No radiographs taken on Western Health’s GE mobile X‐ray machines were included in this study. As this was a clinical study using real, diagnostic radiographs, the authors made some allowance for overlying artefacts or pathology that may obscure landmarks used for image quality scoring, excluding a small number of radiographs, examples of which are shown in Figure 1.

After applying the inclusion and exclusion criteria, there were 26 AP pelvis radiographs acquired at 75 kVp, 30 AP pelvis radiographs acquired at 85 kVp, 22 lateral lumbar spine radiographs acquired at 80 kVp and 31 lateral lumbar spine radiographs acquired at 90 kVp eligible for inclusion. These numbers were rounded to 20 radiographs per group, for even comparisons and ease of image analysis.

### Image analysis

**Figure 2:**
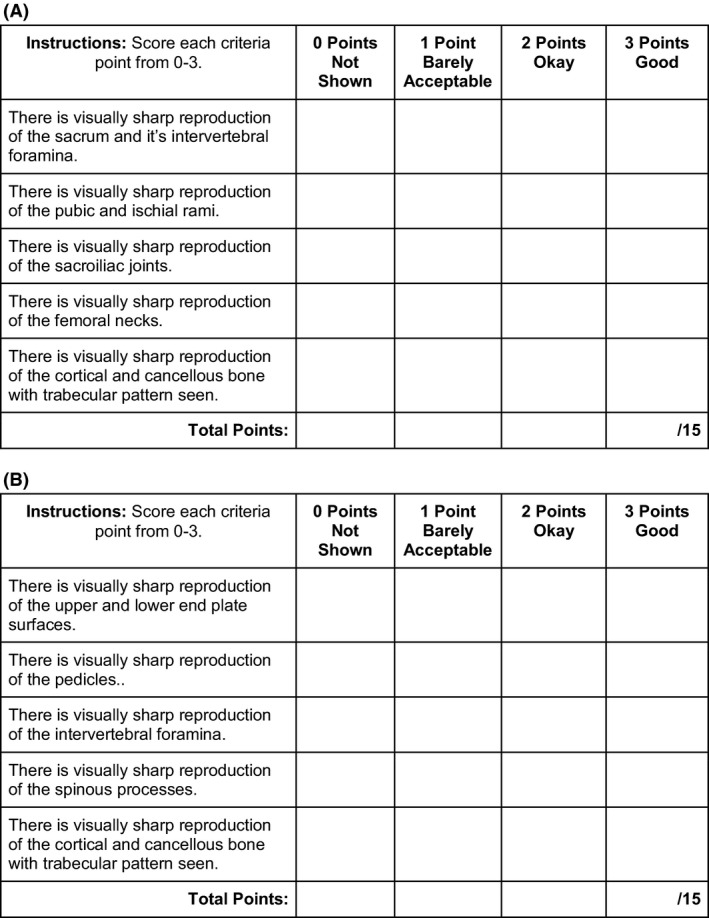
Example VGA Rubric for (A) AP pelvis projection (B) Lateral lumbar spine projection. Derived from the commission of European communities guidelines for image quality criteria for diagnostic radiographic images,^26^ the visual grading analysis rubric was used to quantitatively assess the contrast and image quality of radiographs in this study.

All images were viewed on a Dell P3320 LCD monitor (Dell, Round Rock, USA) with 1680 x 1050‐pixel resolution through the Fujifilm Synapse PACS (Fujifilm Medical Systems, Stamford, USA). The same monitor was used by each reviewer to limit any variance in the display. Radiographs were independently assessed by 5 senior radiographers, each with at least 15 years of clinical experience. Assessors were blinded to the exposure factors used for each image, which were presented in a randomised order. Windowing on PACS was permitted, to mimic conditions of clinical image analysis and critique. Each image was awarded a score from 0 to 15, based on a visual grading analysis (VGA) rubric, shown in Figure 2, that was derived from the Commission of European Communities Guidelines for Image Quality Criteria for Diagnostic Radiographic Images.^26^ These guidelines have been widely used and adapted for image quality assessments in the literature, with a number of the previously mentioned high‐kVp studies adopting these guidelines in some capacity.^8^, ^11^, ^12^, ^14^, ^15^, ^16^

### Data analysis

The non‐parametric Mann–Whitney U‐test was used to determine whether a statistically significant difference between VGA scores existed between the two groups, high and low kVp. This test allowed for a standard z‐score to be derived with a corresponding *P* probability value, through ranking the scores awarded and calculating the rank sum of scores in each group. A z‐score greater than 1.96 shows statistical significance at the 95% confidence interval.^27^

Individual DAP values were used as an estimate of mean patient radiation doses for each projection, in each individual kVp group.

## Results

### VGA results

**Figure 3:**
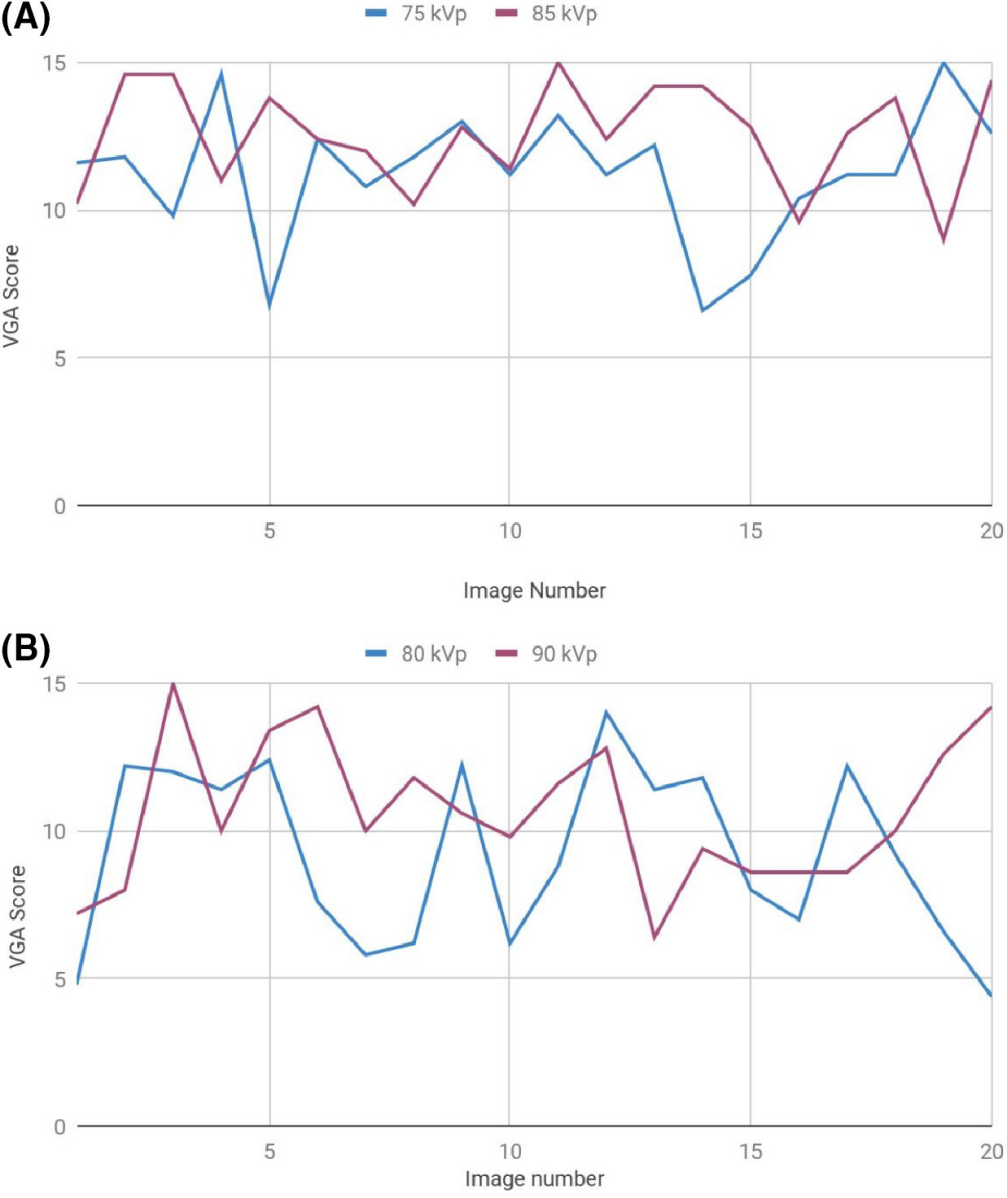
Average VGA scores of (A) AP pelvis images (B) Lateral lumbar spine images. The mean VGA score for each individual radiograph is shown in these graphs. Whilst there is some variance seen, more often than not the higher kVp radiographs (purple lines) show higher VGA scores and therefore better image quality, than the lower kVp group.

**Figure 4:**
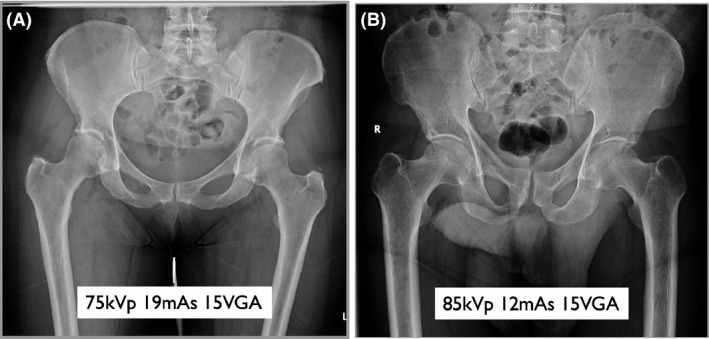
AP pelvis radiographs (A) Low kVp group #19 (B) High kVp group #11. Examples of high VGA scoring AP pelvis radiographs from the low and high kVp groups.

**Figure 5:**
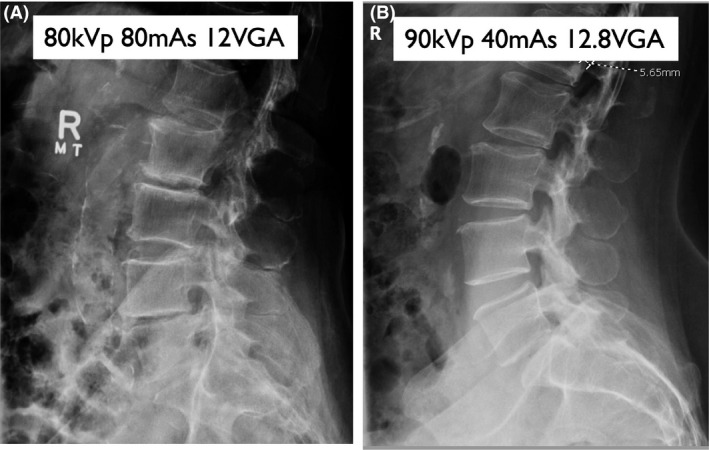
Lateral lumbar spine radiographs (A) Low kVp group #3 (B) High kVp group #12. Examples of high VGA scoring lateral lumbar spine radiographs from the low and high kVp groups

Raw VGA data for the 40 pelvis and 40 lumbar spine radiographs are available in Supplementary Information. The average score for each individual radiograph, for both high and low kVp groups, is presented in Figure 3. High scoring pelvis and lumbar spine images are shown in Figures 4 and 5, respectively, with average VGA scores for reference. The mean VGA score for AP pelvis at 75 kVp was 11.26 and at 85 kVp was 12.55. The mean VGA score for lateral lumbar spines at 80 kVp was 9.23 and at 90 kVp was 10.64. The Mann–Whitney U‐test was applied to the AP pelvis data, low vs high kVp groups, giving a probability value of *P* = 0.06. The lateral lumbar spine data, low vs high kVp groups, gave a probability value of *P* = 0.12. The AP pelvis and lateral lumbar spine data were combined to assess low vs high kVp overall, giving a z‐score of z = 3.1 and probability value of *P* = 0.03.

### DAP results

**Figure 6:**
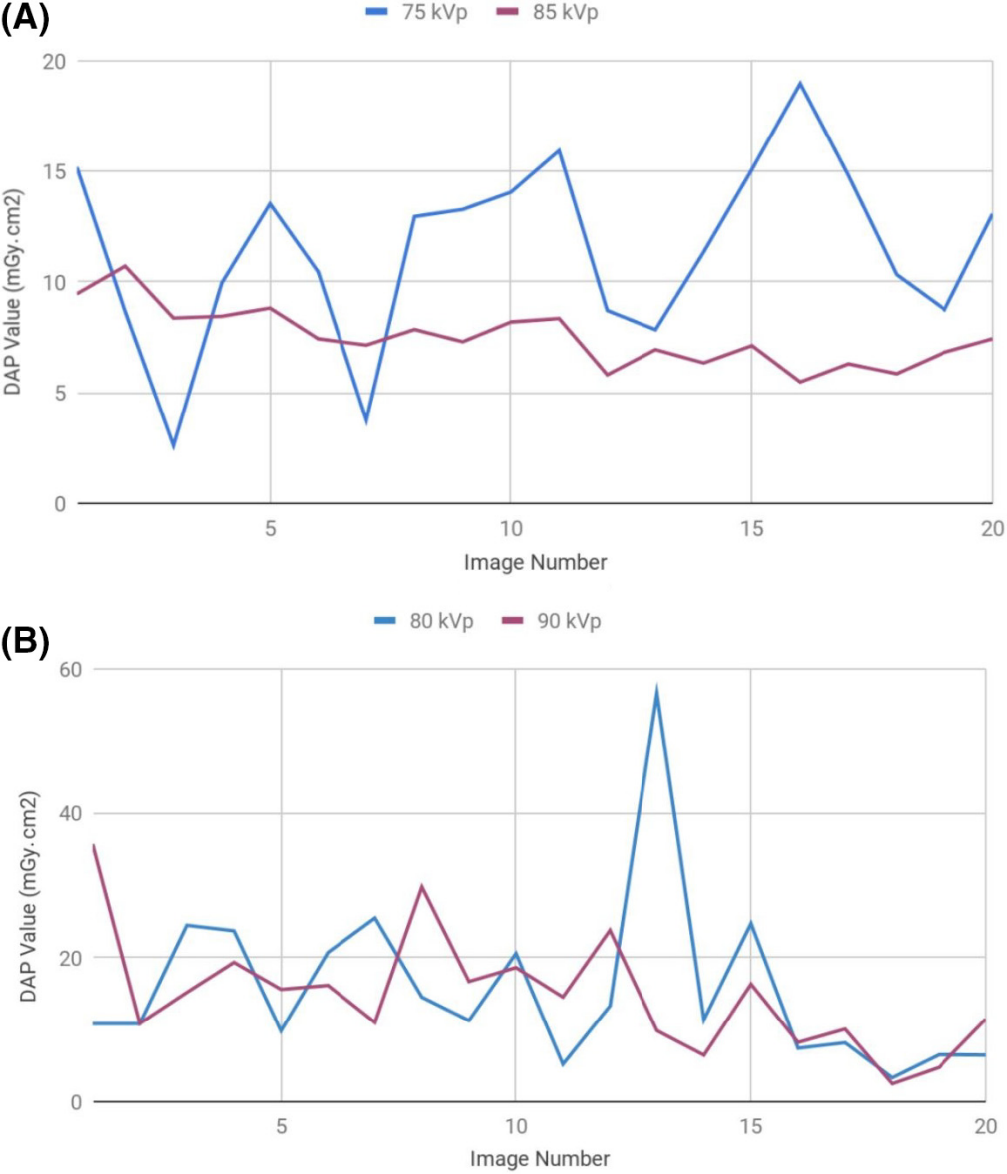
DAP values of (A) AP pelvis images (B) Lateral lumbar spine images. The DAP value represents the radiation dose delivered to the patient in each projection. Reduced radiation doses are seen in the higher kVp groups (purple lines), with more pronounced dose savings seen in the pelvic radiographs

Raw DAP data for the 40 pelvis and 40 lumbar spine radiographs are available in Supplementary Information and are graphed for visual representation in Figure 6. The mean DAP values as calculated for the AP pelvis were 14.06 mGy.cm^2^ at 75 kVp and 7.47 mGy.cm^2^ at 85 kVp. The mean DAP values as calculated for the lateral lumbar spine were 15.76 mGy.cm^2^ at 80 kVp and 14.83mGy.cm^2^ at 90 kVp.

## Discussion

### VGA results

The probability values of *P* = 0.06 and *P* = 0.12, for the AP pelvis and lateral lumbar spine VGA results, respectively, prove that there is no statistically significant difference between the rank sums of the VGA scores from the high and low kVp groups for either projection. These results were in keeping with the hypothesis that increasing kVp would not significantly reduce radiographic image quality, and agree with a number of published materials, that when digital systems are used the image quality and contrast are not heavily reliant on the kVp value.^6^, ^18^, ^19^ Interestingly, the mean VGA scores for the high and low kVp groups showed increased average scores for the higher kVp groups in both projections, by over 1 VGA point. When all data from both projections were combined, a z‐score of 3.1 shows a statistically significant difference between the high and low kVp groups, favouring high kVp.

These results support the findings of de Vries, whose contrast detail phantom imaging also saw slightly higher image quality scores when higher kVp values were used.^4^ Although these results disagree with findings from a number of other studies,^9^, ^14^, ^20^, ^21^, ^22^, ^23^, ^24^ the clinical nature of this study design and the specific projections used may help explain the differences. Both Fauber^9^ and Andria^20^ imaged phantoms (a pelvis phantom and a Leeds Test Object phantom, respectively) in their research in strict laboratory settings, meaning all conditions of image acquisition were kept constant and identical tissue thickness and tissue type was used for all acquisitions. This retrospective, clinical study design did not allow for control over image acquisition conditions, nor was data collected on patient size or thickness. Whilst every effort was made to ensure comparable radiographs were included, the potential variance from a number of influencing factors could explain the discrepancies in results. Whilst Guo’s study was also a clinical study, their research looked into the changes to image quality of paediatric chest radiographs with varying kVp values.^14^ Chest radiography deliberately utilises high kVp values, generally between 110 and 125 kVp, to produce a low contrast radiograph allowing for optimal visualisation of the fine vascular markings of the lungs.^18^ The projections used in the current study are specifically looking at bony anatomy and therefore have different goals to chest imaging (i.e. high contrast required to visualise bony trabecular patterns).

The results from the current study also disagree with studies investigating low‐kVp, variable detector dose techniques, that found improved image quality when lower kVp values were used. Geijer^5^ found improved image quality and lowered effective patient doses when lumbar spine kVp values were reduced from 77 to 60 kVp for the AP projection and from 90 to 77 kVp for the lateral. Kuwahara^24^ assessed image quality and lung lesion visibility on phantom chest radiographs varying from 90 to 140 kVp. Images acquired at 90 kVp were deemed to have superior image quality, with no significant difference seen between lesion visibility. These two low‐kVp studies, however, utilised the more modern concept of variable detector exposure and also adjusted other radiographic factors to achieve these results, with Geijer^5^ increasing the system speed from 400 to 800 and Kuwahara^24^ adding copper filtration.

### DAP results

Overall, for both projections studied, results saw decreased DAP values and therefore decreased patient radiation doses, when the higher kVp values were used. This agrees with and validates the high kVp technique as a dose saving tool on direct digital radiographic systems.^8^, ^9^, ^11^, ^12^, ^13^, ^14^, ^15^, ^16^, ^17^ There were, however, differences in the amount of dose reduction observed between each projection and region of interest. For AP pelvis projections, increasing the kVp from 75 to 85 saw a 46.9% reduction in the mean DAP value. For lateral lumbar spine projections, increasing the kVp from 80 to 90 saw only a 5.9% reduction in the mean DAP value. This difference may be explained by dose saving taper off, as described by Reis;^13^ as kVp values increase the percentage of dose savings detected between each step lessens.

### Study limitations

This study solely focuses on the traditional film/screen concept of a ‘10‐kVp’ rule for constant detector dose and reduced patient doses. This technique is just one of many image quality/dose optimisation methods available in modern digital radiography. As discussed previously, the clinical and retrospective nature of this study design left the authors with no control over specific image acquisition conditions, or information on patient size or thickness. The study therefore relied on radiographic parameters to ensure the radiographs used, and patient thickness for each, were somewhat comparable. The DI value was used as one measure of standardisation; however, many variables can effect its calculation, such as collimation size, centring point and patient thickness. Given that the DI is influenced by collimation, it should be noted that in the case of the AP pelvis radiographs, automated collimation occurs to the 41 × 41 cm detector size and so the collimation is assumed to be the same for each image. This was not the case for the lateral lumbar spine images which were collimated within the dimensions of the detector and therefore present as a limitation of the study.

Although some allowance was made for gross artefacts or pathology, the clinical radiographs used may have had some artefact or pathology (e.g. overlying bowel gas) that made visualisation of relevant bony landmarks difficult, negatively influencing the VGA scores.

Next, no sophisticated radiation dose measurements were taken at the time of image acquisition; dose measurements were taken from the estimated DAP value recorded on the GE system, which will have a margin for error. DAP recordings from the system can be influenced by the collimated area, and this could lead to erroneous readings. As mentioned, the collimated field size for the AP pelvis images was consistent but this is not the case for the lateral lumbar spine images.

Lastly, the monitor used for image reviewing was not a diagnostic standard display used for radiology reporting. This could also have the potential to negatively influence the VGA scores.

### Future directions

To confirm results seen in this retrospective study, further research with larger sample sizes and more observers to score images would be beneficial. Different regions of interest and radiographic projections, especially those that require soft tissue detail, such as abdominal x‐rays, would also be useful to investigate. After the promising VGA results of this study, the possibility of further increase to kVp values, to push the limits of this dose saving phenomenon, could also be considered. With many image quality/dose optimisation techniques available in digital radiography, further research comparing fixed and variable detector exposure methods and their ability to improve diagnostic image quality whilst decreasing patient radiation doses is needed.

## Conclusion

This study allowed for quantifiable assessment of the effect of increasing kVp values on radiographic image quality and assessment of patient radiation doses for x‐ray imaging of the lumbar spine and pelvis in a clinical setting using direct digital detectors. The results supported the hypothesis that increasing kVp values will not significantly reduce image quality. The image quality of all radiographs was diagnostically acceptable, and overall, a reduction in patient radiation dose (in terms of DAP values) was observed when higher kVp values were used. This study successfully validates the high kVp technique as a useful tool for reducing patient radiation doses whilst maintaining high diagnostic image quality for pelvis and lumbar spine imaging.

## Conflict of Interest

The authors declare no conflict of interest.

## Supporting information


**Supplementary Information**. Raw VGA results for (a) AP pelvis images (b) Lateral lumbar spine images. Each radiograph was scored by five individual assessors from 0 to 15 using the VGA rubric for radiographic contrast and image quality.
