# Supplementary material for: An evaluation of the effect of tube potential on clinical image quality using direct digital detectors for pelvis and lumbar spine radiographs
Source: J Med Radiat Sci. 2020 Jun 3;67(4):260–8. doi: 10.1002/jmrs.403 (PMC7753839; doi:10.1002/jmrs.403)
Supplement: Supplementary file 1 — Supplementary Information. Raw VGA results for (a) AP pelvis images (b) Lateral lumbar spine images. Each radiograph was scored by five individual assessors from 0 to 15 using the VGA rubric for radiographic contrast and image quality. [file JMRS-67-260-s001.docx]

***Supplementary Information - Raw VGA Results for (a) AP pelvis images (b) Lateral lumbar spine images. Each radiograph was scored by five individual assessors from 0-15 using the VGA rubric for radiographic contrast and image quality.***

***a.***

| **Pelvis Image** | **kVp/mAs** | **DAP** mGy.cm^2^ | **VGA Assessor 1** | **VGA Assessor 2** | **VGA Assessor 3** | **VGA Assessor 4** | **VGA Assessor 5** |
| --- | --- | --- | --- | --- | --- | --- | --- |
| 1 | 75/20 | 15.19 | 10 | 13 | 14 | 10 | 11 |
| 2 | 75/16 | 8.676 | 11 | 12 | 14 | 10 | 12 |
| 3 | 75/15 | 2.62 | 8 | 10 | 10 | 11 | 10 |
| 4 | 75/16 | 9.96 | 15 | 15 | 15 | 14 | 14 |
| 5 | 75/20 | 13.52 | 8 | 8 | 5 | 7 | 6 |
| 6 | 75/20 | 10.46 | 12 | 13 | 12 | 12 | 13 |
| 7 | 75/20 | 3.764 | 10 | 11 | 11 | 9 | 13 |
| 8 | 75/25 | 12.96 | 13 | 13 | 12 | 11 | 10 |
| 9 | 75/25 | 13.28 | 12 | 12 | 12 | 14 | 15 |
| 10 | 75/20 | 14.06 | 12 | 9 | 12 | 10 | 13 |
| 11 | 75/20 | 15.94 | 15 | 14 | 13 | 10 | 14 |
| 12 | 75/16 | 8.717 | 10 | 9 | 10 | 13 | 14 |
| 13 | 75/20 | 7.849 | 12 | 12 | 11 | 13 | 13 |
| 14 | 75/25 | 11.36 | 8 | 5 | 9 | 6 | 5 |
| 15 | 75/25 | 15.09 | 11 | 9 | 8 | 5 | 6 |
| 16 | 75/25 | 18.97 | 12 | 10 | 12 | 9 | 9 |
| 17 | 75/20 | 14.81 | 15 | 9 | 9 | 11 | 13 |
| 18 | 75/20 | 10.34 | 11 | 10 | 13 | 8 | 14 |
| 19 | 75/19 | 8.757 | 15 | 15 | 15 | 15 | 15 |
| 20 | 75/25 | 13.08 | 14 | 12 | 12 | 12 | 13 |
| 1 | 85/12 | 8.468 | 10 | 10 | 8 | 9 | 14 |
| 2 | 85/12 | 10.72 | 15 | 14 | 15 | 15 | 14 |
| 3 | 85/12 | 8.375 | 15 | 15 | 15 | 13 | 15 |
| 4 | 85/12 | 8.456 | 13 | 11 | 10 | 12 | 9 |
| 5 | 85/12 | 8.827 | 13 | 13 | 14 | 15 | 14 |
| 6 | 85/12 | 7.44 | 11 | 10 | 13 | 15 | 13 |
| 7 | 85/12 | 7.154 | 13 | 12 | 11 | 14 | 11 |
| 8 | 85/12 | 7.859 | 8 | 9 | 8 | 11 | 15 |
| 9 | 85/12 | 7.306 | 13 | 12 | 10 | 14 | 15 |
| 10 | 85/12 | 8.197 | 14 | 11 | 7 | 12 | 13 |
| 11 | 85/12 | 8.356 | 15 | 15 | 15 | 15 | 15 |
| 12 | 85/9 | 5.814 | 12 | 13 | 11 | 13 | 13 |
| 13 | 85/10 | 6.952 | 12 | 14 | 15 | 15 | 15 |
| 14 | 85/9 | 6.349 | 14 | 14 | 13 | 15 | 15 |
| 15 | 85/10 | 7.13 | 13 | 12 | 10 | 14 | 15 |
| 16 | 85/9 | 5.49 | 10 | 6 | 9 | 11 | 12 |
| 17 | 85/9 | 6.308 | 12 | 15 | 10 | 13 | 13 |
| 18 | 85/9 | 5.862 | 13 | 14 | 12 | 15 | 15 |
| 19 | 85/10 | 6.834 | 7 | 7 | 9 | 12 | 10 |
| 20 | 85/11 | 7.441 | 14 | 14 | 14 | 15 | 15 |

***b.***

| **Lumbar Image** | **kVp/ mAs** | **DAP** mGy.cm^2^ | **VGA Assessor 1** | **VGA Assessor 2** | **VGA Assessor 3** | **VGA Assessor 4** | **VGA Assessor 5** |
| --- | --- | --- | --- | --- | --- | --- | --- |
| 1 | 80/82 | 10.83 | 4 | 8 | 4 | 0 | 8 |
| 2 | 80/82 | 10.83 | 7 | 14 | 14 | 14 | 12 |
| 3 | 80/80 | 24.46 | 11 | 14 | 12 | 13 | 10 |
| 4 | 80/86 | 23.69 | 10 | 13 | 9 | 14 | 11 |
| 5 | 80/100 | 9.818 | 12 | 13 | 10 | 15 | 12 |
| 6 | 80/63 | 20.7 | 5 | 9 | 7 | 8 | 9 |
| 7 | 80/80 | 25.47 | 5 | 4 | 7 | 4 | 9 |
| 8 | 80/84 | 14.49 | 4 | 6 | 7 | 8 | 6 |
| 9 | 80/76 | 11.26 | 10 | 14 | 14 | 14 | 9 |
| 10 | 80/61 | 20.55 | 5 | 7 | 4 | 6 | 9 |
| 11 | 80/80 | 5.2 | 6 | 9 | 9 | 10 | 10 |
| 12 | 80/80 | 13.29 | 14 | 15 | 15 | 14 | 12 |
| 13 | 80/60 | 56.61 | 10 | 14 | 7 | 15 | 11 |
| 14 | 80/63 | 11.38 | 10 | 15 | 9 | 13 | 12 |
| 15 | 80/80 | 24.72 | 6 | 8 | 7 | 13 | 6 |
| 16 | 80/80 | 7.412 | 4 | 9 | 3 | 12 | 7 |
| 17 | 80/63 | 8.175 | 13 | 14 | 11 | 15 | 8 |
| 18 | 80/63 | 3.324 | 7 | 12 | 6 | 13 | 8 |
| 19 | 80/100 | 6.52 | 5 | 8 | 4 | 9 | 7 |
| 20 | 80/68 | 6.468 | 4 | 5 | 1 | 6 | 6 |
| 1 | 90/50 | 35.69 | 6 | 5 | 8 | 10 | 7 |
| 2 | 90/40 | 10.85 | 9 | 1 | 9 | 9 | 12 |
| 3 | 90/50 | 15.16 | 15 | 15 | 15 | 15 | 15 |
| 4 | 90/32 | 19.32 | 10 | 12 | 10 | 7 | 11 |
| 5 | 90/37 | 15.58 | 12 | 14 | 14 | 14 | 13 |
| 6 | 90/39 | 16.13 | 14 | 15 | 15 | 14 | 13 |
| 7 | 90/32 | 10.99 | 9 | 8 | 14 | 10 | 9 |
| 8 | 90/40 | 29.77 | 12 | 10 | 12 | 14 | 11 |
| 9 | 90/33 | 16.67 | 12 | 13 | 8 | 6 | 14 |
| 10 | 90/47 | 18.6 | 9 | 10 | 10 | 8 | 12 |
| 11 | 90/40 | 14.51 | 9 | 11 | 10 | 15 | 13 |
| 12 | 90/40 | 23.77 | 11 | 15 | 8 | 15 | 15 |
| 13 | 90/32 | 9.848 | 6 | 5 | 7 | 5 | 9 |
| 14 | 90/45 | 6.451 | 10 | 8 | 6 | 9 | 14 |
| 15 | 90/49 | 16.31 | 6 | 6 | 13 | 7 | 11 |
| 16 | 90/49 | 8.219 | 5 | 9 | 15 | 7 | 7 |
| 17 | 90/50 | 10.06 | 6 | 10 | 5 | 12 | 10 |
| 18 | 90/31 | 2.487 | 4 | 11 | 12 | 13 | 10 |
| 19 | 90/29 | 4.762 | 10 | 15 | 11 | 14 | 13 |
| 20 | 90/32 | 11.45 | 12 | 15 | 15 | 14 | 15 |
